# Supplementary material for: Mutational fitness landscapes of diverse human influenza H1N1 neuraminidases
Source: bioRxiv. 2026 Oct 8:2026.10.07.757144. Preprint. [Version 1] doi: 10.64898/2026.10.07.757144 (PMC13692211; doi:10.64898/2026.10.07.757144)
Supplement: Supplementary file 1 [file NIHPP2026.10.07.757144v1-supplement-4.pdf]

bioRxiv preprint doi: https://doi.org/10.64898/2026.10.07.757144; this version posted October 8, 2026. The copyright holder for this preprint (which was not certified by peer review) is the author/funder, who has granted bioRxiv a license to display the preprint in perpetuity. It is made available under a CC-BY 4.0 International license.

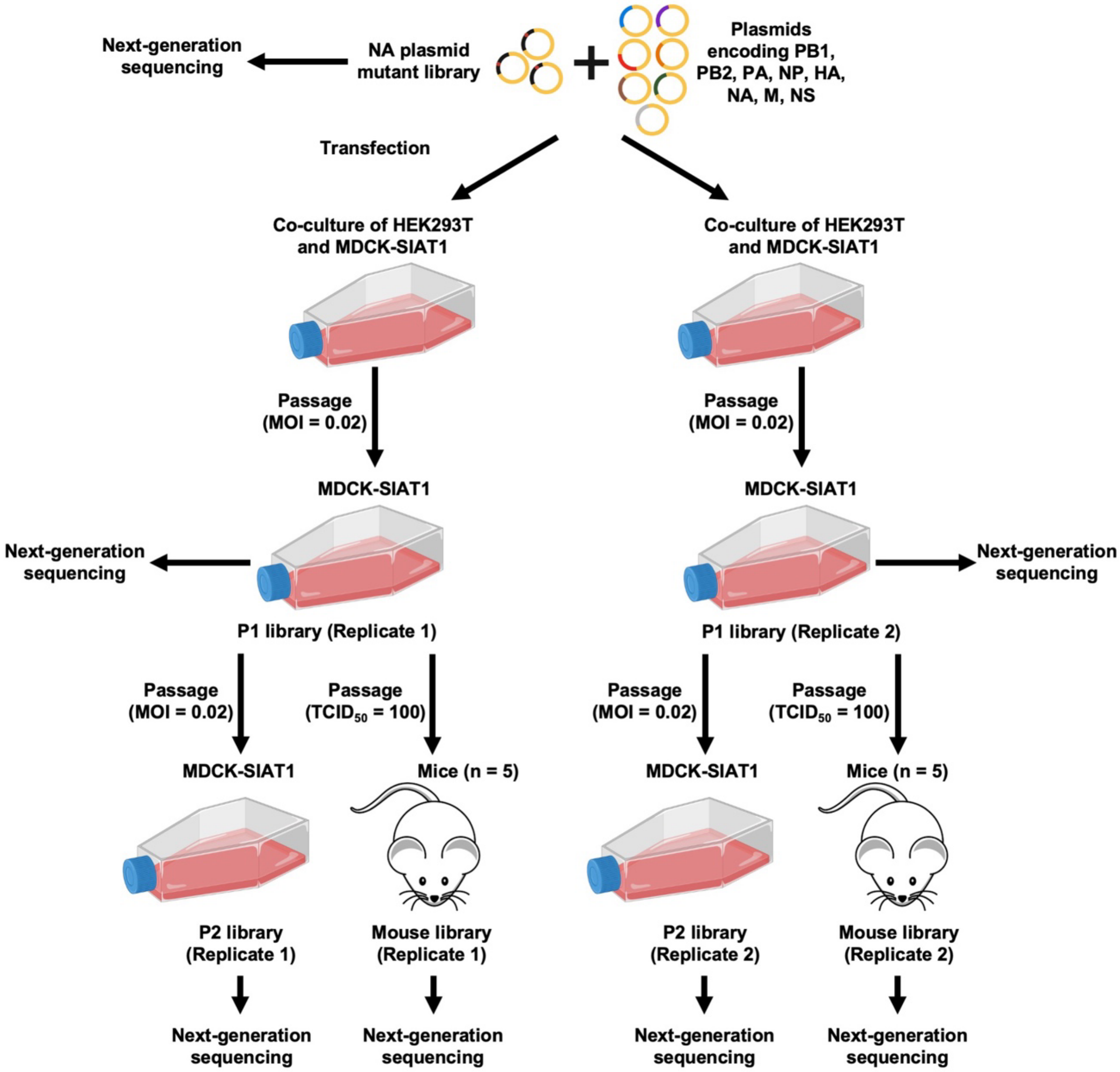


**Figure S1. Experimental design.** The PR8 NA virus mutant library was rescued by transfecting the PR8 NA plasmid mutant library along with plasmids encoding seven internal segments from A/Puerto Rico/8/1934 (H1N1) into a co-culture of HEK293T cells and MDCK-SIAT1 cells. The PR8 NA virus mutant library was then passaged in MDCK-SIAT1 cells at a multiplicity of infection (MOI) of 0.02 to generate the post-passaged P1 mutant library. The P1 library was further passaged either in MDCK-SIAT1 cells at MOI 0.02 (P2 library) or in mice at 100 $TCID_{50}$ (mouse library). A biological replicate of transfection and passaging was performed using the same NA plasmid mutant library. For each mouse biological replicate, lungs from five mice were pooled. The NA plasmid mutant library and both biological replicates of the P1, P2, and mouse libraries were analyzed by next-generation sequencing.

bioRxiv preprint doi: https://doi.org/10.64898/2026.10.07.757144; this version posted October 8, 2026. The copyright holder for this preprint (which was not certified by peer review) is the author/funder, who has granted bioRxiv a license to display the preprint in perpetuity. It is made available under a CC-BY 4.0 International license.

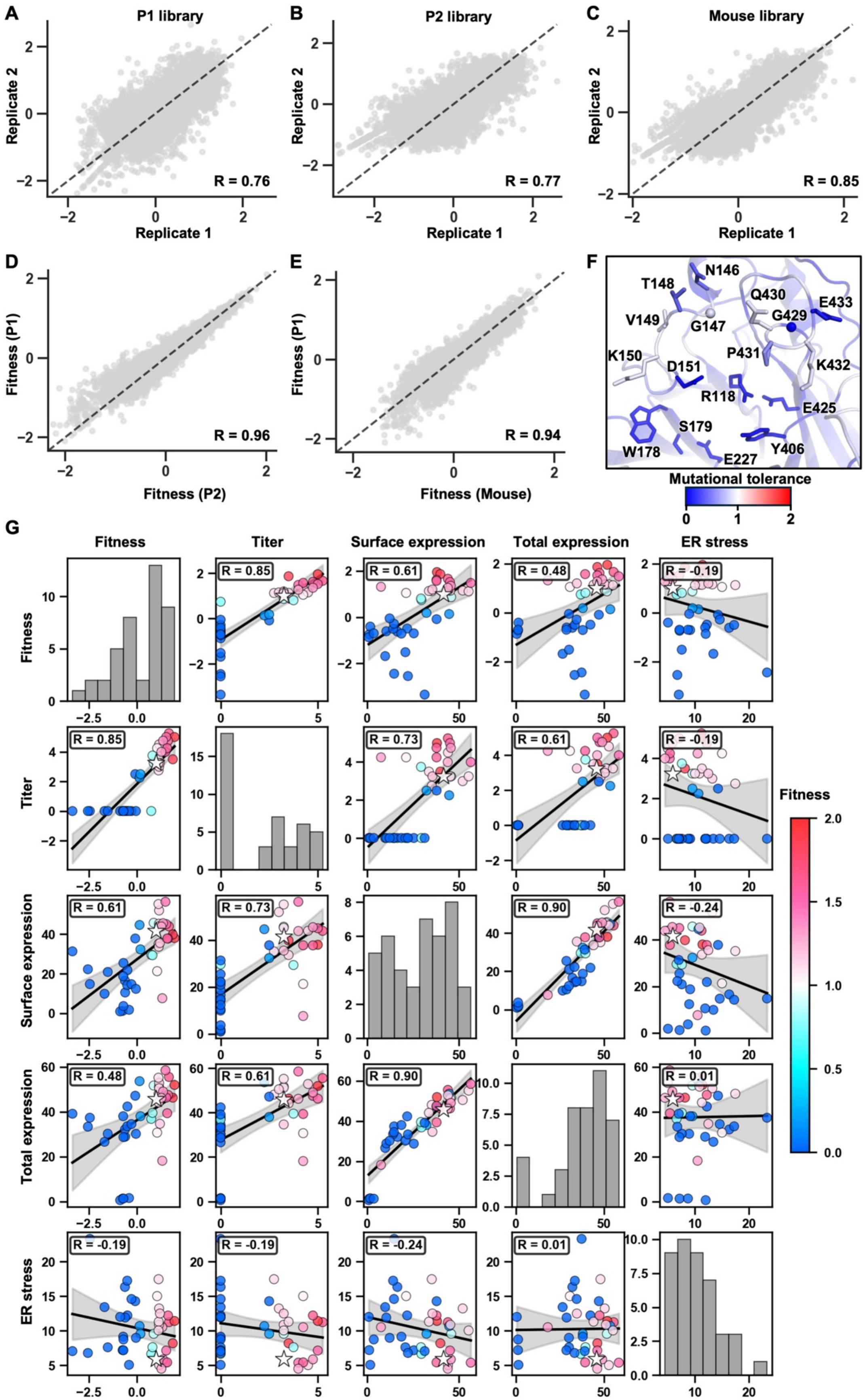

bioRxiv preprint doi: https://doi.org/10.64898/2026.10.07.757144; this version posted October 8, 2026. The copyright holder for this preprint (which was not certified by peer review) is the author/funder, who has granted bioRxiv a license to display the preprint in perpetuity. It is made available under a CC-BY 4.0 International license.

**Figure S2. Reproducibility and quality of N1/PR8 deep mutational scanning data. (A-C)** Correlation of fitness scores for individual mutations between two biological replicates for **(A)** P1 library, **(B)** P2 library, and **(C)** mouse library is shown as a scatterplot. **(D and E)** Scatterplot showing the correlation of fitness scores for individual mutations between P1 library and **(D)** P2 library, or **(E)** mouse library. **(F)** Mutational tolerance across NA residues of N1/PR8 P1 library mapped onto the NA structure from H1N1 A/Brevig Misson/1/1918 (PDB: 8E6K)[1]. Catalytic residues (R118 and Y406), framework residues (W178, S179, E277, and E425), 150 loop (G147 and K150) and 430-loops (Q430 and K432) are labeled. **(G)** Scatterplots showing the relationships among fitness score, viral titer, NA surface expression, NA total expression, and ER stress. Individual data points are color mapped, where red and blue indicate beneficial and deleterious mutations, respectively. WT is represented by a star. **(A-E and G)** The Pearson correlation coefficient (R) is indicated.

bioRxiv preprint doi: https://doi.org/10.64898/2026.10.07.757144; this version posted October 8, 2026. The copyright holder for this preprint (which was not certified by peer review) is the author/funder, who has granted bioRxiv a license to display the preprint in perpetuity. It is made available under a CC-BY 4.0 International license.

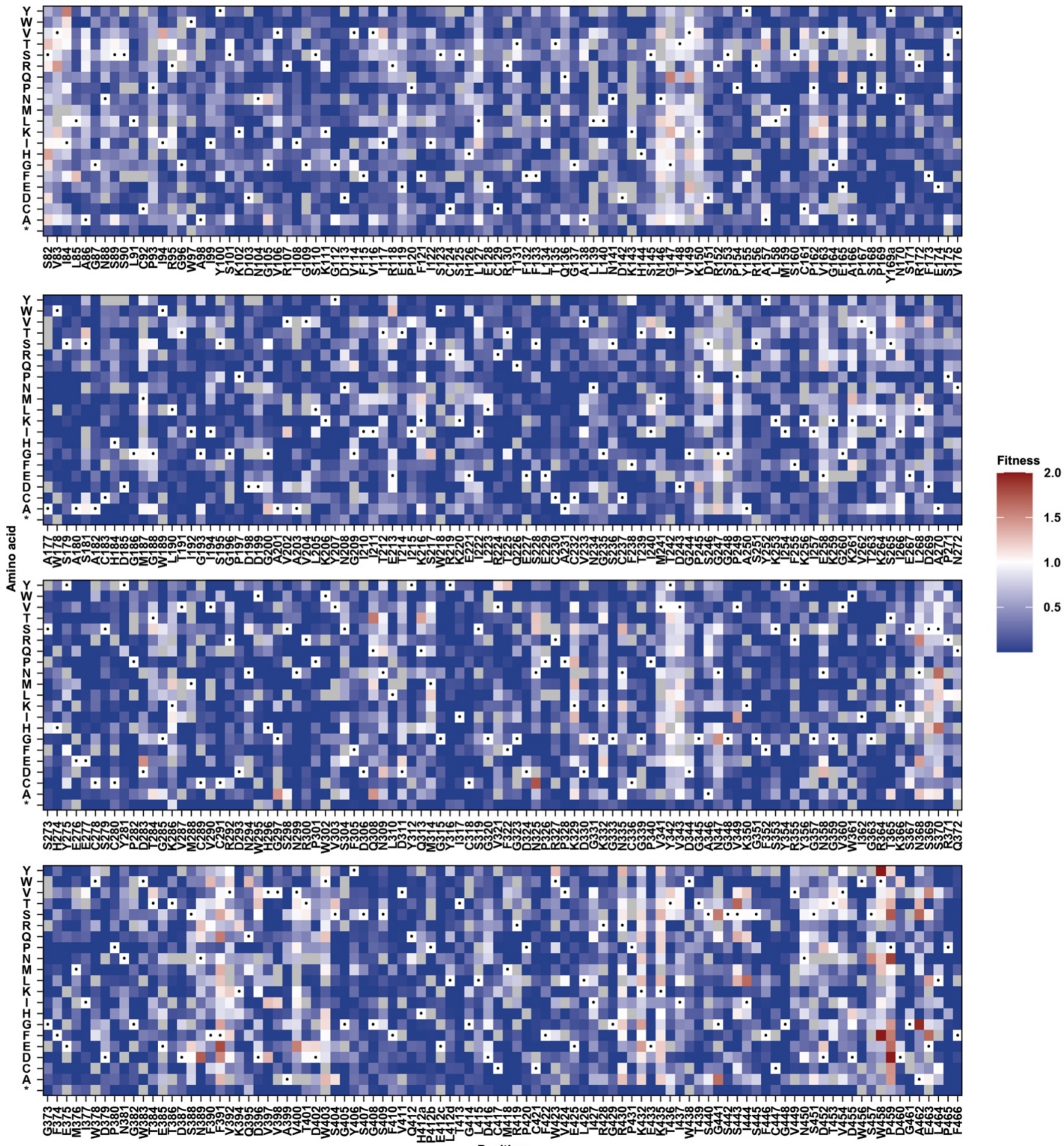


**Figure S3. Deep mutational scanning of N1/Mar43 NA.** The replication fitness effects of individual mutations in the N1/Mar43 NA library were measured by deep mutational scanning and fitness scores are shown as a heatmap. Wild-type (WT) amino acids are indicated by a black circle. Mutations in gray were excluded in our data analysis due to low input count. Stop codon is annotated as *.

bioRxiv preprint doi: https://doi.org/10.64898/2026.10.07.757144; this version posted October 8, 2026. The copyright holder for this preprint (which was not certified by peer review) is the author/funder, who has granted bioRxiv a license to display the preprint in perpetuity. It is made available under a CC-BY 4.0 International license.

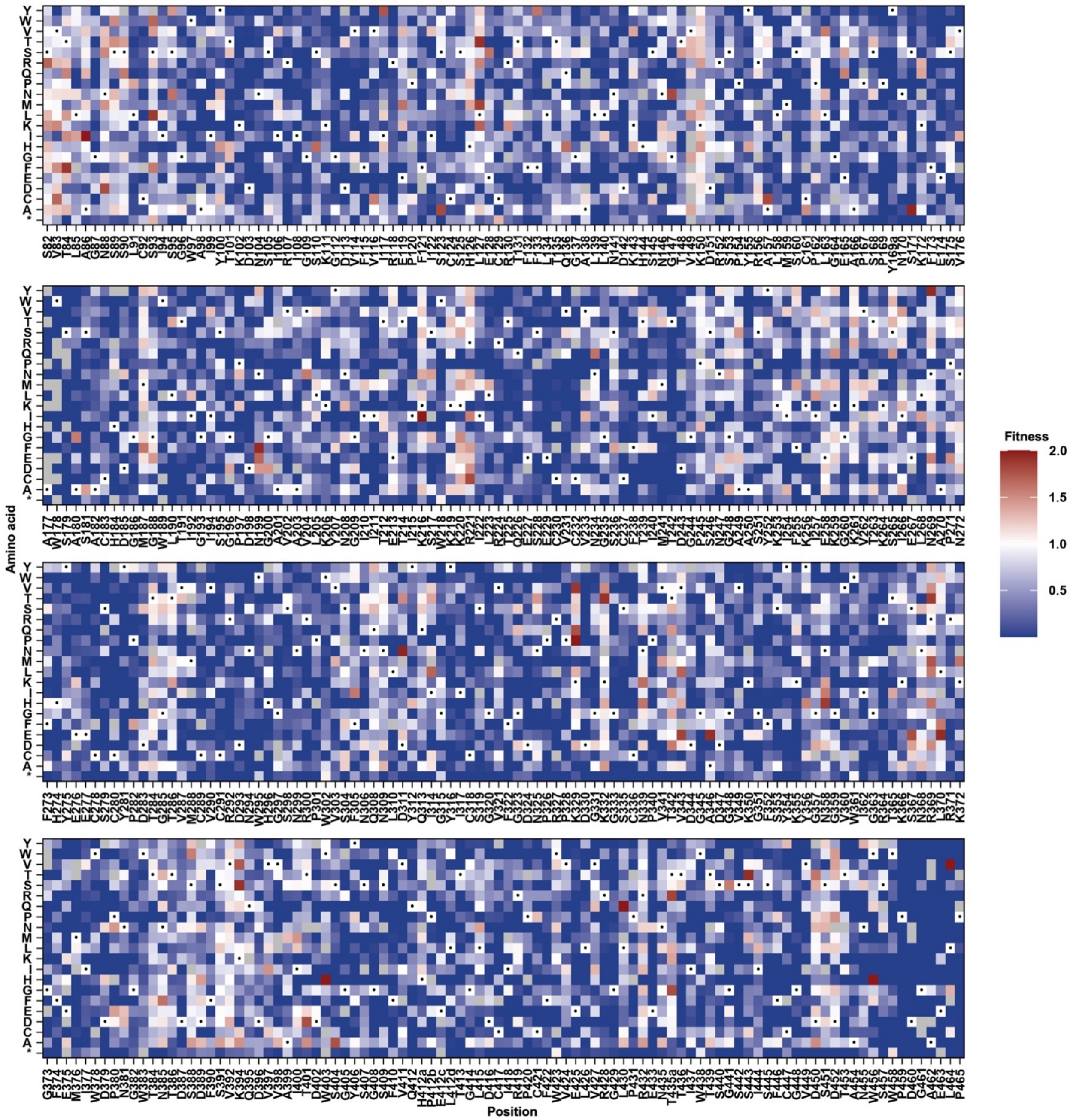


**Figure S4. Deep mutational scanning of N1/Geo06 NA.** The replication fitness effects of individual mutations in the N1/Geo06 NA library were measured by deep mutational scanning and fitness scores are shown as a heatmap. WT amino acids are indicated by a black circle. Mutations in gray were excluded in our data analysis due to low input count. Stop codon is annotated as *.

bioRxiv preprint doi: https://doi.org/10.64898/2026.10.07.757144; this version posted October 8, 2026. The copyright holder for this preprint (which was not certified by peer review) is the author/funder, who has granted bioRxiv a license to display the preprint in perpetuity. It is made available under a CC-BY 4.0 International license.

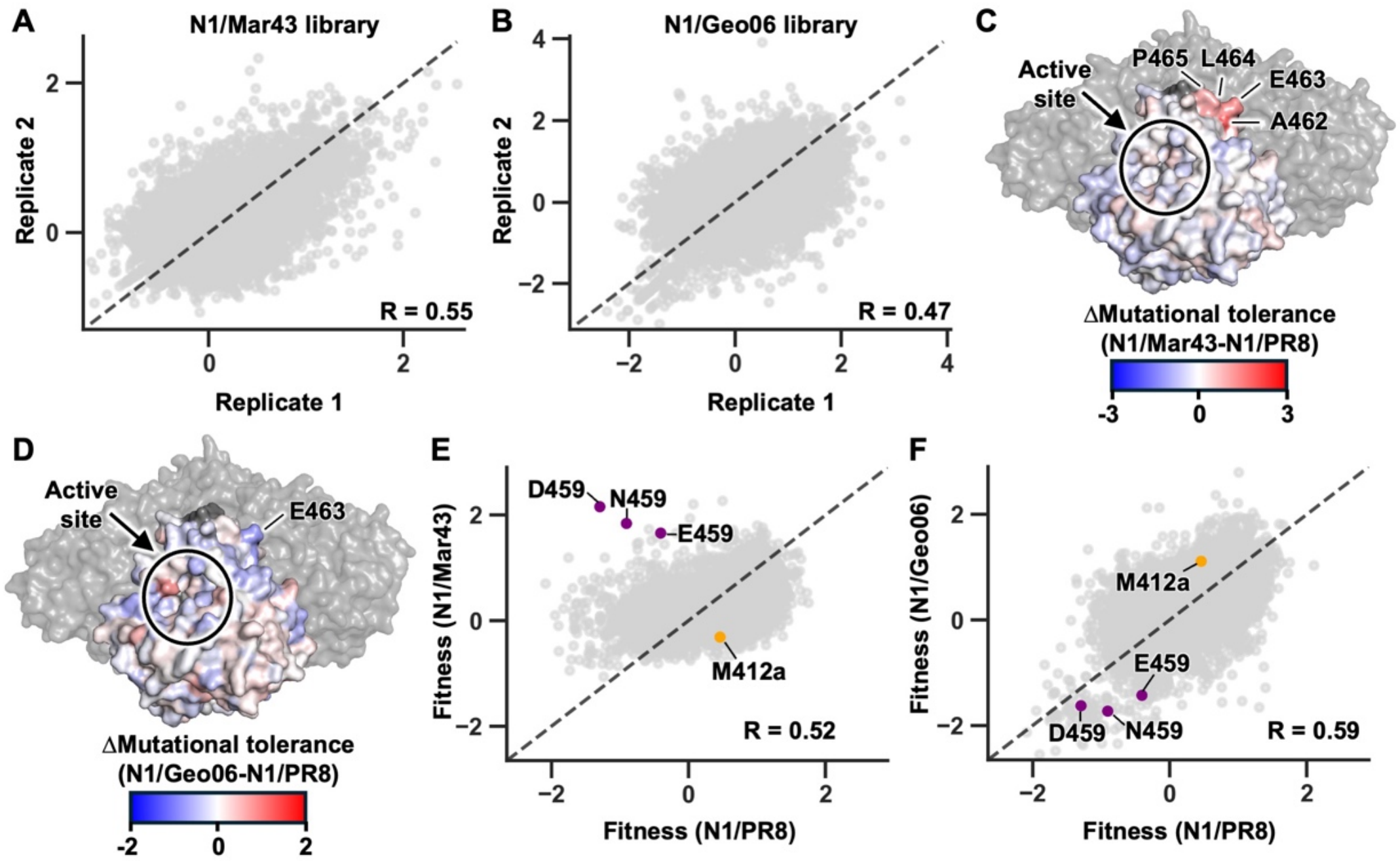


**Figure S5. Reproducibility of the deep mutational scanning experiments and comparison of mutational effects between different H1N1 NAs. (A and B)** Scatterplots showing the correlation of mutational fitness profiles between two biological replicates for **(A)** N1/Mar43 and **(B)** N1/Geo06. Each data point represents one mutation. The Pearson correlation coefficient (R) is indicated. **(C and D)** Difference of mutational tolerance between for **(C)** N1/Mar43 and N1/PR8 and **(D)** N1/Geo06 and N1/PR8 mapped onto the crystal structure of N1/Mar43 NA. One protomer is displayed in surface representation with residues colored according to mutational tolerance, while the remaining three protomers are shown in gray. Active site is circled. **(E and F)** Scatterplots showing the correlation of fitness scores for individual mutations between **(E)** N1/Mar43 and N1/PR8 and **(F)** N1/Geo06 and N1/PR8. H412aM, and P459D/N/E are indicated in orange and purple, respectively. Pearson correlation coefficient (R) is indicated.

bioRxiv preprint doi: https://doi.org/10.64898/2026.10.07.757144; this version posted October 8, 2026. The copyright holder for this preprint (which was not certified by peer review) is the author/funder, who has granted bioRxiv a license to display the preprint in perpetuity. It is made available under a CC-BY 4.0 International license.

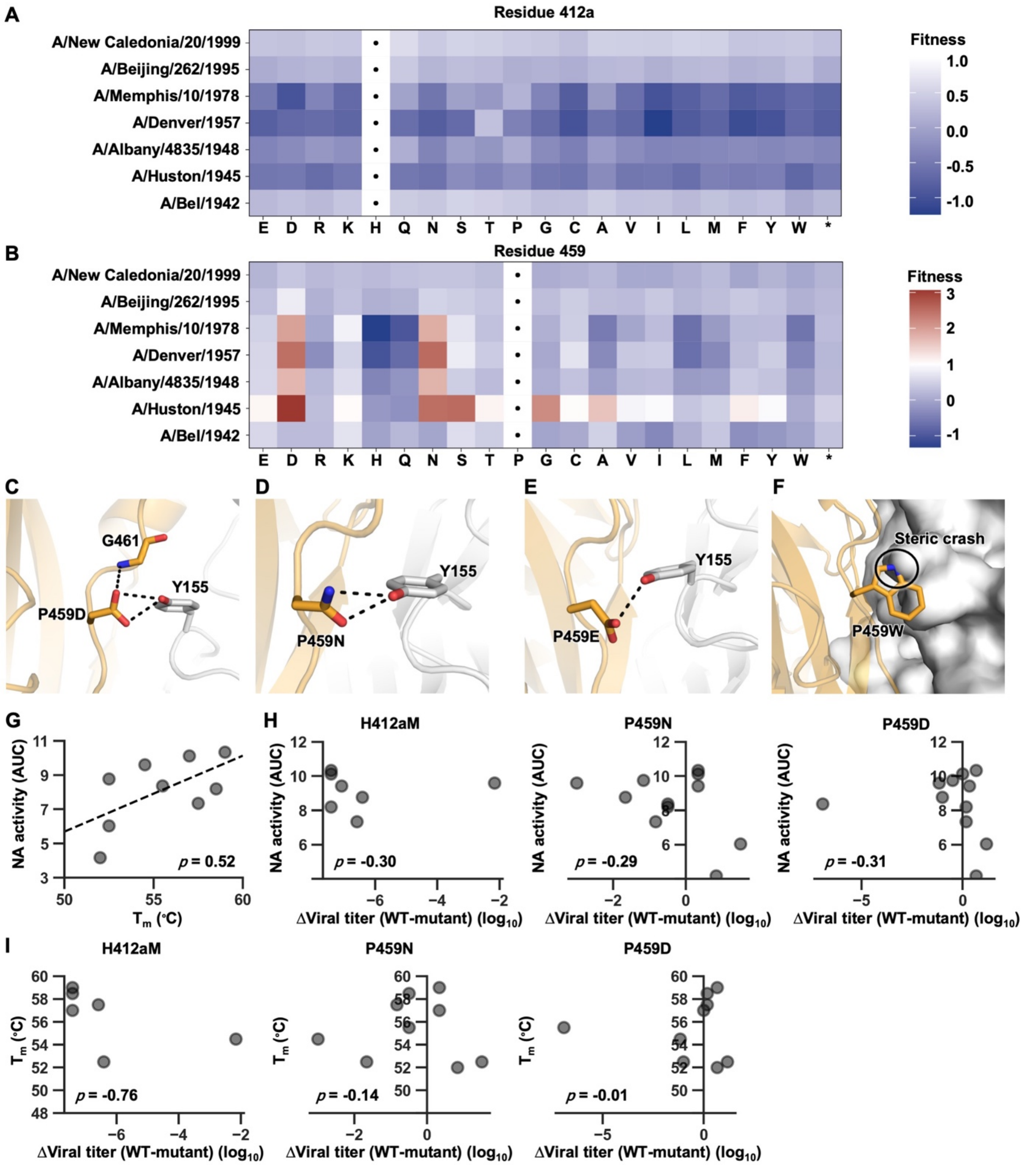


**Figure S6. Mutational fitness effect at residues 412a and 459. (A and B)** The fitness scores of mutations at **(A)** H412a and **(B)** P459 across different human H1N1 strains are shown as a heatmap. WT amino acids are indicated by a black circle. Stop codon is annotated as *. **(C-F)** Interactions between neighboring residues with P459D/N/E/W is shown. **(G)** Correlation between NA enzymatic activity and thermostability. NA activity was quantitated by area under curve (AUC). The temperature corresponding to the lowest point of the first derivative -d(RFU)/dT was determined to be the melting temperature ($T_m$). Of note, thermostability data for A/Bel/1942 NA

bioRxiv preprint doi: https://doi.org/10.64898/2026.10.07.757144; this version posted October 8, 2026. The copyright holder for this preprint (which was not certified by peer review) is the author/funder, who has granted bioRxiv a license to display the preprint in perpetuity. It is made available under a CC-BY 4.0 International license.

and N1/Mar43 NA are excluded from the analysis due to the presence of two distinct transitions in the thermal shift assay. **(H and I)** Correlation between **(H)** WT NA enzymatic activity or **(I)** thermostability and difference in viral titer between WT and H412aM or P459N/D. **(G-I)** The Spearman's rank correlation coefficient ($\rho$) is indicated.

bioRxiv preprint doi: https://doi.org/10.64898/2026.10.07.757144; this version posted October 8, 2026. The copyright holder for this preprint (which was not certified by peer review) is the author/funder, who has granted bioRxiv a license to display the preprint in perpetuity. It is made available under a CC-BY 4.0 International license.

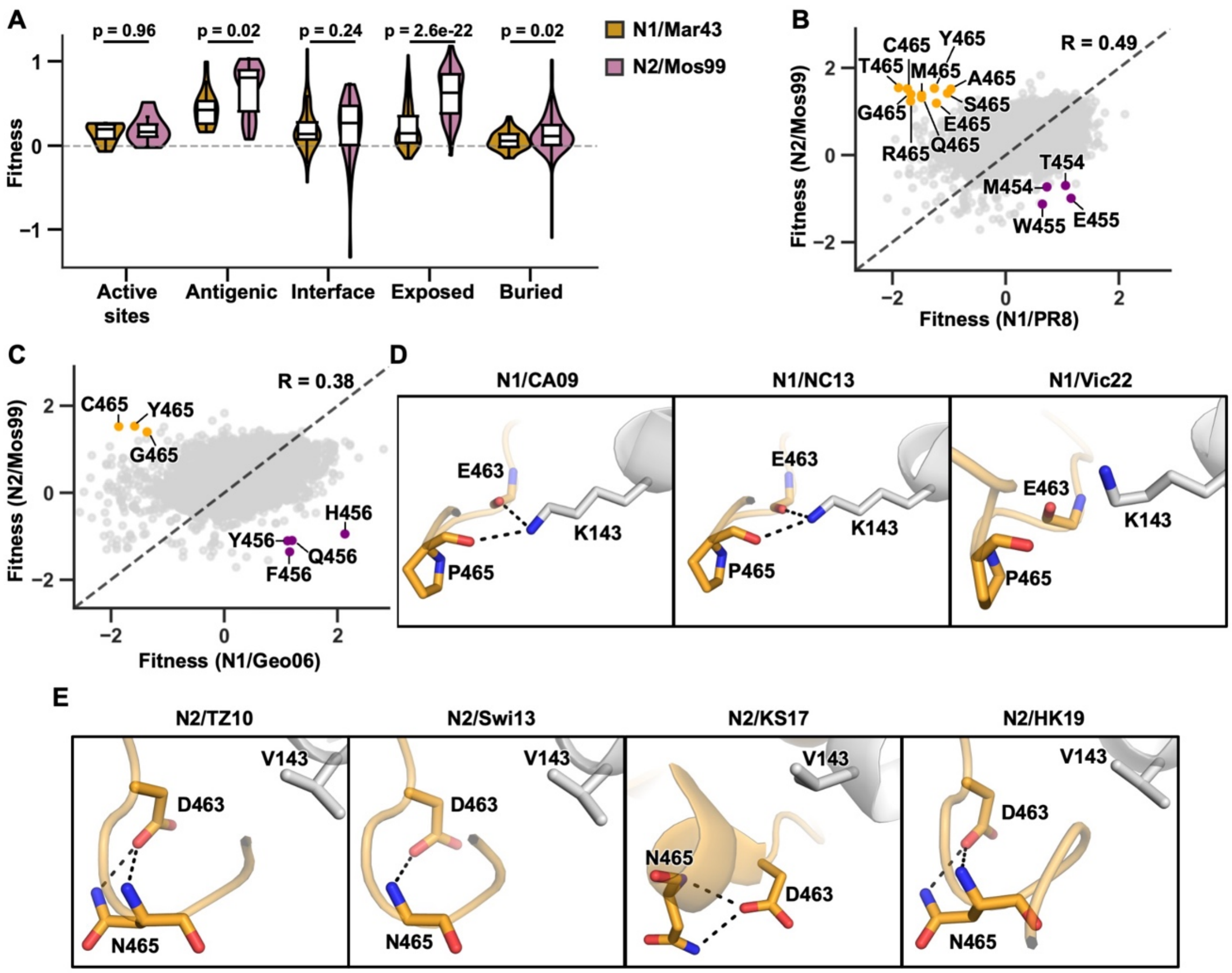


**Figure S7. Comparison between N1 and N2 NAs. (A)** Violin plots showing the distribution of mean fitness scores for active site, N1/N2 antigenic site, interface, exposed and buried residues. Yellow indicates the N1/Mar43 library, and pink indicates the N2/Mos99 library. Statistical significance was analyzed by two-sided Wilcoxon rank-sum tests, with p-values were adjusted using the Benjamini-Hochberg procedure. **(B and C)** Scatterplot showing the correlation of mutational fitness profiles between **(B)** N2/Mos99 and N1/PR8 as well as **(C)** N2/Mos99 and N1/Geo06. Each data point represents one mutation. Beneficial mutants in N2/Mos99 and N1/PR8 or N1/Geo06 is indicated in orange and purple, respectively. Pearson correlation coefficient (R) is indicated. **(D)** Interactions between P465 and neighboring residues in different N1 are shown. H1N1 A/California/04/2009 (N1/CA09, PDB: 9O9V)[2], H1N1 A/North Carolina/07/2013 (N1/NC13, PDB: 9EWQ)[3], H1N1 A/Victoria/4897/2022 (N1/Vic22, PDB: 9DPC)[4]. **(E)** Interactions between N465 and neighboring residues in different N2 are shown. H3N2 A/Tanzania/205/2010 (N2/TZ10, PDB: 8G3Q)[5], H3N2 A/Switzerland/9715293/2013 (N2/Swi13, PDB: 8ZYX)[6], H3N2 A/Kansas/14/2017 (N2/KS17, PDB: 8ZR4)[7] and H3N2 A/Hong Kong/2617/2019 (N2/HK19, PDB: 8G3V)[5]. Black dashed lines represent H-bonds.

bioRxiv preprint doi: https://doi.org/10.64898/2026.10.07.757144; this version posted October 8, 2026. The copyright holder for this preprint (which was not certified by peer review) is the author/funder, who has granted bioRxiv a license to display the preprint in perpetuity. It is made available under a CC-BY 4.0 International license.

## Supplementary References

1. Hansen, L., McMahon, M., Turner, H.L., Zhu, X., Turner, J.S., Ozorowski, G., Stadlbauer, D., Vahokoski, J., Schmitz, A.J., and Rizk, A.A. (2023). Human anti-N1 monoclonal antibodies elicited by pandemic H1N1 virus infection broadly inhibit HxN1 viruses in vitro and in vivo. Immunity *56*, 1927-1938. e1928.
2. Lederhofer, J., Borst, A.J., Nguyen, L., Gillespie, R.A., Williams, C.J., Walker, E.L., Raab, J.E., Yap, C., Ellis, D., and Creanga, A. (2025). Structural convergence and water-mediated substrate mimicry enable broad neuraminidase inhibition by human antibodies. Nature Communications *16*, 7068.
3. Pascha, M.N., Ballegeer, M., Roelofs, M.C., Meuris, L., Albulescu, I.C., van Kuppeveld, F.J., Hurdiss, D.L., Bosch, B.-J., Zeev-Ben-Mordehai, T., and Saelens, X. (2024). Nanoparticle display of neuraminidase elicits enhanced antibody responses and protection against influenza A virus challenge. npj Vaccines *9*, 97.
4. Madsen, A., Okba, N.M., Pholcharee, T., Matz, H.C., Lv, H., Ibanez Trullen, M., Zhou, J.Q., Turner, J.S., Schmitz, A.J., and Han, F. (2025). Identification of a seasonal influenza vaccine–induced broadly protective neuraminidase antibody. Journal of Experimental Medicine *222*, e20241930.
5. Momont, C., Dang, H.V., Zatta, F., Hauser, K., Wang, C., di Iulio, J., Minola, A., Czudnochowski, N., De Marco, A., and Branch, K. (2023). A pan-influenza antibody inhibiting neuraminidase via receptor mimicry. Nature *618*, 590-597.
6. Lv, K., Li, X., Zhu, X., Cai, J.-P., Liu, S., Sun, Y., Liu, L., Cai, X., Cao, R., and Xu, M. (2025). Broad neuraminidase antibodies confer protection against seasonal and avian influenza viruses. Nature Communications *16*, 7103.
7. Wang, X., Kong, H., Chu, B., Yang, Q., Lin, C., Liu, R., Chen, C., Gao, Y., Wang, G., and Wang, D. (2025). Identification of a broad-inhibition influenza neuraminidase antibody from pre-existing memory B cells. Cell Host & Microbe *33*, 151-166. e158.
